# Antimicrobial PMMA Bone Cement Containing Long Releasing Multi-Walled Carbon Nanotubes

**DOI:** 10.3390/nano12081381

**Published:** 2022-04-18

**Authors:** Yazan Al Thaher, Raida Khalil, Sharif Abdelghany, Mutaz S. Salem

**Affiliations:** 1 Faculty of Pharmacy, Philadelphia University, Amman 19392, Jordan; salem@philadelphia.edu.jo; 2 Department of Biotechnology and Genetic Engineering, Faculty of Science, Philadelphia University, Amman 19392, Jordan; r_khalil@philadelphia.edu.jo; 3 School of Pharmacy, The University of Jordan, Amman 11942, Jordan; s.abdelghany@ju.edu.jo; 4 Faculty of Pharmacy, Jordan University of Science and Technology, Irbid 22110, Jordan

**Keywords:** PMMA, bone cement, carbon nanotubes, gentamicin, antimicrobial, cytotoxicity, compression strength

## Abstract

Prosthetic joint infections (PJIs) ensued from total joint replacement (TJR) pose a severe threat to patients that involve poor health outcomes, severe pain, death (in severe cases), and negative influence patients’ quality of life. Antibiotic-loaded bone cement (ALBC) is frequently used for the prevention and treatment of PJI. This work aims to study gentamicin release from carbon nanotubes (CNTs) incorporated in polymethyl methacrylate (PMMA) bone cement to prolong release over several weeks to provide prophylaxis from PJIs after surgery. Different CNT concentrations were tested with the presence of gentamicin as a powder or preloaded onto carboxyl functionalized CNTs. The different types of bone cement were tested for drug release, mechanical properties, water uptake, antimicrobial properties, and cytocompatibility with human osteoblast cells (MTT, LDH, alizarin red, and morphology). Results showed prolonged release of gentamicin from CNT-loaded bone cements over several weeks compared to gentamicin-containing bone cement. Additionally, the presence of CNT enhanced the percentage of gentamicin released without adversely affecting the nanocomposite mechanical and antimicrobial properties needed for performance. Cytotoxicity testing showed non-inferior performance of the CNT-containing bone cement to the equivalent powder containing cement. Therefore, the developed nanocomposites may serve as a novel PMMA bone cement to prevent PJIs.

## 1. Introduction

Total joint replacement (TJR) is performed mainly in end-stage arthritis and for hip and knee replacements, with aging and obesity as risk factors [1]. Prosthetic joint infections (PJI) can occur in patients following TJR, mostly in the first few months after surgery [2,3]. PJIs can pose a severe threat to patients’ health that can involve severe pain and death (in severe cases), and which can negatively influence patients’ quality of life [4,5]. Moreover, PJIs inflict a substantial economic burden on healthcare systems, as their management requires repeated surgeries and longer hospitalization duration [6].

PMMA bone cement is frequently used in cemented TJRs, where PMMA can fix the implant in place and release loaded antibiotics. Antibiotic-loaded bone cements (ALBCs) are frequently employed for the prevention and treatment of PJI after joint replacement [7]. The most commonly used antibiotics in ALBCs are aminoglycosides, particularly gentamicin [8]. Aminoglycosides have broad-spectrum antibacterial action and can bear high temperatures caused by the exothermic PMMA polymerization reaction. Gentamicin is available as a powder, either premixed with the powder part of the cement formulation or added as an off-label formulation [9].

Despite the wide use of ALBCs, many concerns have been raised about the release profile of antibiotics [10,11,12]. In particular, the release profile is described by a burst release for hours or a few days after surgery, which falls below the inhibitory levels needed to prevent infections [13]. A prolonged-release profile is needed for the prophylaxis from both early and delayed infections that can occur in the first few weeks to months after surgery [14]. Furthermore, less than 10% of the loaded antibiotic is released, and the bulk remains trapped within the cement matrix [15,16]. Therefore, improving local antibiotic release for ALBCs is essential for the management of PJIs, without affecting other properties required for the performance and function of the bone cement.

Many studies have been aimed at improving the release profile for gentamicin from PMMA bone cement by nanoparticle incorporation, including silica nanoparticles [17,18], liposomes [19], clay nanotubes [20], hydroxyapatite, and multi-walled carbon nanotubes (CNTs) [21]. Among these nano-delivery systems, CNTs have the ability to enhance the thermal, mechanical and structural properties of PMMA cement [22,23,24]. Furthermore, CNTs containing cements have been reported to have satisfactory cytocompatibility and biological properties [25,26,27,28]. However, the effect of CNT on the delivery of antibiotics from bone cements has not been adequately studied, because most of the studies were focused on improving cytocompatibility and mechanical properties of the PMMA cement without studying the effect on antibiotic release. To the best of our knowledge, Shen et al. (2016) is the only study that reported enhanced elution of gentamicin from CNT-containing PMMA cement; however, the high percentage of CNT (5.3%) impaired the cement mechanical properties [21]. In this study, lower concentrations of CNT were studied for optimizing antibiotic release without adversely affecting other properties needed of cement performance.

This study aims to prolong gentamicin release from CNT-incorporated PMMA bone cement for several weeks to provide prophylaxis from PJIs after surgery. Different CNT concentrations were tested with the presence of gentamicin as a powder or preloaded onto carboxyl functionalized CNTs by wet impregnation. The different cements were tested for drug release, mechanical performance, water uptake, antimicrobial properties, and cytocompatibility with human osteoblasts (MTT, LDH, alizarin red, and morphology). It was hypothesized that CNT concentration can be optimized for enhancing gentamicin release without adversely affecting the PMMA nanocomposite properties needed for performance.

## 2. Materials and Methods

### 2.1. Chemicals

The carboxyl functionalized CNT powder, containing a 10% carboxylic acid concentration, was used in this study (Sigma-Aldrich code: 755125, supplier: Nanocyl Inc., Sambreville, Belgium). The average diameter of the CNT was 9.5 nm and length was 1.5 μm. Gentamicin sulphate, phosphate buffer solution (PBS) tablets, glutaraldehyde, o-phthaldialdehyde reagent solution (OPA), dimethyl sulfoxide, alizarin red S, β-glycerol phosphate, ascorbic acid, and dexamethasone were purchased from Sigma-Aldrich, Gillingham, UK. The bone cement brand used was Cemex^®^ (Tecres^®^ SpA, Verona, Italy) and was used based on supplier guidelines.

### 2.2. Preparation of Gentamicin-Loaded CNT

Gentamicin was loaded into the CNTs by wet impregnation, as previously described (with some modifications) [21]. Typically, 0.20 g of gentamicin was dissolved in 3 mL deionized water. 0.30 g of CNT powder was then impregnated with the gentamicin solution under stirring for 24 h. The mixture was dried under vacuum for 48 h at room temperature. The dried gentamicin-loaded CNTs were manually ground to fine powder, and the required quantity of nanoparticles was mixed with the powder part of the commercial cement.

### 2.3. Characterization for CNT and Gentamicin-Loaded CNTs

Transmission electron microscope (TEM)

TEM images were taken by a Morgagni FEI 268 (FEI Company, Eindhoven, The Netherlands) microscope at 60 kV equipped with Megaview 3 digital camera, at 100,000× magnification. One drop of nanoparticles suspension was placed on a Formvar/carbon-coated (200 mesh) copper grid and allowed to evaporate at room temperature.

Fourier-transformed infrared (FTIR)

The FTIR spectra were measured using a Thermo Nicolt NEXUS 670 FT-IR spectrometer (Thermo Nicolet Corporation, Madison, WI, USA) in the range 4000–400 cm^−1^ with a resolution of 4 cm^−1^. The filler was mixed with potassium bromide (KBr) after drying, then, ground and pressed into thin sheets for testing.

Zeta potential measurements

Dynamic light scattering was utilized to measure the electrophoretic mobility for the CNTs by a Malvern Zetasizer Nano ZS (Malvern Instruments, Malvern, Worcestershire, UK). Smoluchowski model was used to convert the measured electrophoretic mobility to zeta potential value (ζ). For each measurement, the CNTs were dispersed in buffer solutions at a concentration of 1 mg/mL at pH ranging from 4 to 7. Each zeta value represented an average for three different measurements.

### 2.4. Bone Cement Preparation

Four cement formulations were studied: Cemex with 3% gentamicin powder, Cemex with 3% of gentamicin-loaded CNTs (prepared in Section 2.2), Cemex with 0.3% CNT and 3% gentamicin powder, and Cemex with 1% CNT and 3% gentamicin powder. Cement preparation was performed according to the ISO5833:2002 (Implants for Surgery—Acrylic Resin Cements) and manufacturer’s instructions [29]. The constituents of the bone cement were stored as per manufacturer’s guidelines (8–15 °C for the liquid in the dark and 20–25 °C for the powder). Before mixing, the constituents were conditioned to room temperature (22 °C) for 2 h.

Both components (powder and liquid) were manually mixed in a polypropylene bowl using a polypropylene spatula for 1 min. Then the cement dough was poured into a polytetrafluoroethylene (PTFE) mold with specific shapes and dimensions suitable for the chosen tests. The mold was then clamped with two PTFE film-covered steel endplates. After 2 h, the samples were removed from the mold and cured at 23 °C for 24 ± 2 h. Finally, 320-grit silicon carbide paper was used to sand down the samples, smoothing the edges to have the correct dimensions.

### 2.5. Gentamicin Release Quantification

Cylindrical samples with 10 mm height and 6 mm diameter were prepared using a suitable PTFE mold. The samples (weighed 0.40 ± 0.01 g) were incubated in 3 mL PBS buffer (pH 7.4) at 37 °C. The buffer was replaced every day to achieve sink conditions to keep the concentration of the released drug insignificant compared to saturation solubility [17,18].The samples were stored in a refrigerator (2–8 °C) for 3 days before analysis. Three replicates were used for each cement type. The amount of gentamicin released was measured using o-phthaldialdehyde reagent through fluorescence spectroscopy, which gives a fluorogenic product through the reaction with the amino groups of gentamicin [30]. In a black 96-well plate, 100 μL of release medium was added with (100 μL) o-phthaldialdehyde reagent and (100 μL) isopropanol. The plate was incubated for 30 min at room temperature in the dark, and then fluorescence determination was performed (λ excitation = 340 nm, λ emission = 450 nm) by a FL ×800 fluorescence microplate reader (BIO-TEK instruments, Winooski, VT, USA). Each time point was an average of 3 samples. For all black 96-well plates, three standard antibiotic solutions with known concentrations (0–100 μg/mL) were measured concurrently with release samples to create a calibration curve.

The release graphs were plotted both as cumulative concentration released and percentage gentamicin released. One of the drawbacks for PMMA bone cement is that less than 10% of the loaded antibiotic is released, and the bulk remains trapped within the cement matrix [15,16]. Therefore, it is important to plot the release as a percentage to have an idea about the total amount of drug released and the amount of drug that stays inside the cement. Additionally, it is important to plot gentamicin as cumulative concentration released, because the antimicrobial action for aminoglycoside antibiotics is concentration dependent, and high peak to minimum inhibitory concentration ratios could reduce the emergence of resistant mutants [31].

The gentamicin-loaded CNT were dispersed (10 mg in 1 mL) in PBS (pH 7.4). Gentamicin release was investigated at 37 °C for samples in Eppendorfs. Release samples were taken every 24 h by replacing all release media with a fresh PBS media. Eppendorfs were centrifuged before replacing media to prevent withdrawal of CNTs.

### 2.6. Mechanical Testing

Compressive testing was conducted according to the ISO 5833:2002 [29], where cylindrical shaped samples were used (diameter and height are 6 mm and 12 mm, respectively). The machine used was a Shimadzu AGX-V (SHIMADZU corporation, Kyoto, Japan) with a software package (TRAPEZIUM™ X-V) at 20 mm/min crosshead speed. Compressive strength was performed after 3 months of sample aging in PBS at 37 °C.

### 2.7. Water Uptake Testing

The cement specimens (6 mm diameter and 10 mm height) were incubated in 3 mL PBS at 37 °C for 30 days. In the first 2 weeks, the samples were weighed every day. Then, they were weighed every three days [17,18]. Water uptake was determined by dividing weight gain by the initial mass of the sample, and samples were tested in triplicate.

### 2.8. Agar Diffusion Assay

Cylindrical cement samples 10 mm diameter by 2 mm height were prepared as previously described in the bone cement preparation section. *Staphylococcus aureus* (*S. aureus*, NCIMB 9518) was cultured in brain heart infusion (BHI) agar for 18–24 h at 37 °C. The inoculum was spread across a BHI Petri dish using a sterile cotton swab. The Petri dish was turned 60 degrees, and the process was repeated for full coverage. A 10 μg gentamicin disc (Oxoid, Southampton, UK) was placed on the Petri dish as control, and pressure was applied to the top of the disc to ensure complete surface contact. The Petri dish was then incubated at 37 °C for 24 h. After that, the zones of inhibition around the gentamicin disc and samples were measured. Images of the zones of inhibition were taken and analyzed using ImageJ^®^ software (Available online: https://sourceforge.net/projects/x264vfw/files/x264vfw64/ (accessed on 25 March 2022)). Zone of inhibition was calculated as the zone’s radius minus the sample’s radius (the experiment was repeated in triplicate, *n* = 3) [19].

### 2.9. Cytotoxicity Analysis

Human dental fibroblasts (HDFs) were gifted from the faculty of Medicine, University of Jordan. HDFs were maintained in complete medium including Dulbecco’s Modified Eagle’s Medium (DMEM, Hyclone, Locan, UT, USA) supplemented with 10% fetal bovine serum (FBS, Welgene, Daejeon, Korea) and 1% *v*/*v* of a solution of 100 U/mL penicillin and 100 µg/mL streptomycin (Sigma-Aldrich, Gillingham, UK) in a 5% CO_2_ humidified incubator at 37 °C. When the confluence reached 70%, HDFs were sub-cultured with a split ratio of 1:8. HDFs conducted in this study were between passage 3 and 5 in all investigations with the cement specimen’s disc (diameter and height of 10 and 5 mm, respectively).

In vitro differentiation of HDFs

HDFs were seeded at passage 3 in a 24-well plate at 10^4^ cells/well density and incubated for 48 h in a complete medium. For osteogenic differentiation, the medium was replaced with osteogenic medium including complete medium supplemented with 10 mM β-glycerol phosphate, 50 µg/mL ascorbic acid, and 100 nM dexamethasone. The osteogenic medium was replaced every 2 days and incubated for a further 24 days [32].

MTT Assay

MTT reagent (Thermo fisher scientific, Paisley, UK) was used to determine cell viability. Each cement specimen was incubated in growth complete medium in a 24-well plate containing 1 × 10^5^ of differentiated osteoblast cells per well for 7 days at 37 °C in a humidified atmosphere with 5% CO_2_. MTT tests were performed after incubation for 1, 2, 4, and 7 days. For each test, the medium present in the well was replaced with phenol free medium and MTT reagent (5 mg/mL). Then, the plate was incubated for a further 24 h at 37 °C. The medium was then removed, dimethyl sulfoxide was added in each well, and the plates were incubated for 10 min. Finally, the dissolved formazan solution was transferred from each well into a 96-well plate for absorbance determination by a microplate reader (Multiscan sky, Thermo Fisher Scientific, Vantaa, Finland) at λ = 560 nm. The results were plotted as percentage viability compared to the cells only control.

LDH (lactate dehydrogenase) measurement

An in vitro Toxicology Assay Kit, LDH-based (Roche, Mannheim, Germany) was utilized to study the viability of cells according to the manufacturer’s protocols. Osteoblast cells were grown in a 24-well plate and as described above (In vitro Differentiation of HDFs). The released LDH was quantified in the media before and after the addition of cell lysis solution (total LDH). Cell viability was determined according to the following equation:

$$Viability\;\left(\%\right)=\frac{\left(Total\;cells-Dead\;cells\right)\;}{Total\;cells\;}\times 100\%$$

Total and released LDH were determined as optical density (OD), at λ = 490 nm, after correcting the reading by a negative control.

Detection of calcium deposits (mineralization)

For the mineralization assay, a 24-well plate containing 1 × 10^5^ of osteoblast cells was incubated with cement specimens at 37 °C for 7 days in a humidified atmosphere with 5% CO_2_. The medium in each well was replaced with glutaraldehyde 10% (*v*/*v*), and the plates were then incubated for 15 min and washed three times with deionized water. Next, alizarin red S 1% (*w*/*v*) was added to each well, and the plates were incubated for 20 min. After washing three times with deionized water, acetic acid 10% (*v*/*v*) was added to each well, and the plates were incubated for a further 30 min. Finally, the solution from each well was moved in the 96-well plate and analyzed using a microplate reader (Multiscan sky, Thermo Fisher Scientific, Vantaa, Finland) at 450 nm. All stained images were evaluated and pictured under a microscope (20× magnification) (Nikon Eclipse TS100, Tokyo, Japan). The results were plotted as percentage viability compared to the cells only control.

### 2.10. Statistical Analysis

Experimental data were presented as mean ± standard deviation (SD) for at least three values. A one-way analysis of variance (ANOVA) was performed to assess the significance between different groups. Statistical significance was conducted at a 95% confidence level (*p* < 0.05). All analyses were run using PRISM^®^9.0 software (GraphPad Software Inc., San Diego, CA, USA).

## 3. Results

### 3.1. Characterization of CNT and Gentamicin-Loaded CNT

Specific characterization conducted by Nanocyl Inc., Sambreville, Belgium, demonstrated the basic properties of the purchased carboxyl functionalized MWCNT powder (Table 1) as mentioned in the certificate of analysis [33].

**Figure 1:**
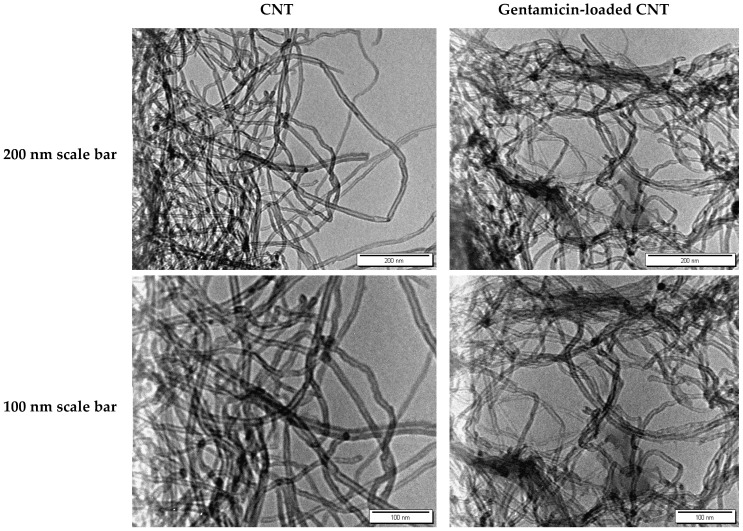
TEM images for CNTs (**left**) and gentamicin-loaded CNTs (**right**).

The TEM images for the CNTs and gentamicin-loaded CNTS are shown in Figure 1.

**Figure 2:**
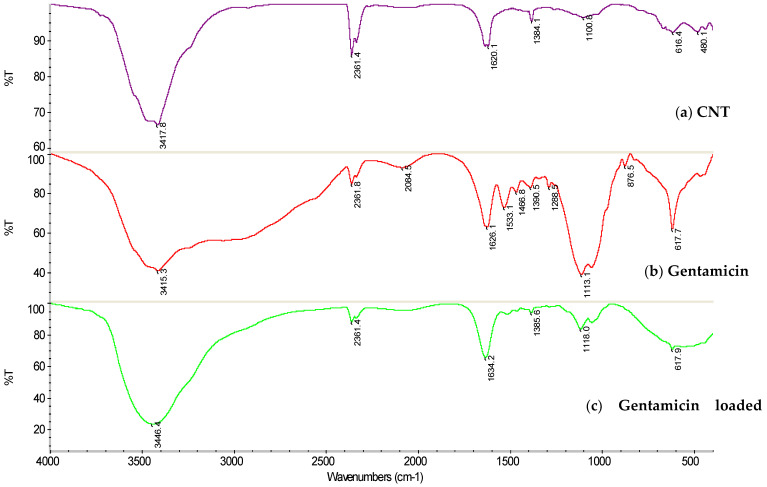
FTIR spectra for (**a**) CNTs, (**b**) gentamicin, and (**c**) gentamicin-loaded CNTs.

The FTIR spectra are shown in Figure 2. A broad diffuse band in the scope of 3650–2500 cm^−1^ designate the gentamicin molecules. The adsorption bands near 1626 cm^−1^ and 3415 cm^−1^ are related to the bending of N–H bond of the primary and secondary amines in gentamicin [34]. The N–H bending vibration peaks at 1634 cm^−1^ and 3446 cm^−1^ in the gentamicin-loaded CNT reflect the presence of gentamicin and clearly show that gentamicin molecules were incorporated in the CNT. The characteristic peak of CNT–COOH can be found at 3417 cm^−1^, which belongs to the OH stretching mode, and the peak near 1100 cm^−1^ belongs to the C–O stretching mode [35]. In the infrared curve of gentamicin-loaded CNT, the characteristic peaks of pure CNT, –NH_2_ of gentamicin could still be found, and the two peak patterns are shifted to 3446 cm^−1^, which could be attributed to electrostatic interaction.

**Figure 3:**
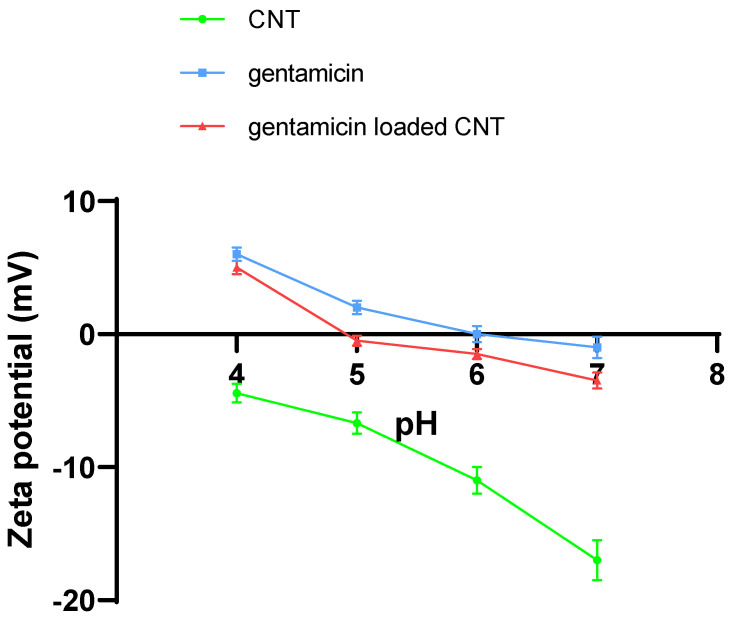
Zeta potentials of CNT, gentamicin, and gentamicin-loaded CNT at different pH values ranging from 4 to 7 (mean ± SD, *n* = 3).

Zeta potential measurements were performed for the CNT, gentamicin, and gentamicin-loaded CNTs (Figure 3). The zeta potential for the pure CNTs was negative and reached −17 mV at pH 7 because of the presence of carboxylate groups. Gentamicin had a small zeta potential close to zero, because it is a weak polyelectrolyte with only five ionizable amino groups that become protonated, giving a positive charge. Gentamicin-loaded CNT zeta potential was close to zero because of the neutralizing effect of the electrostatic interaction between the amino group of gentamicin and the carboxylate group on CNT.

### 3.2. Gentamicin Release from CNTs

**Figure 4:**
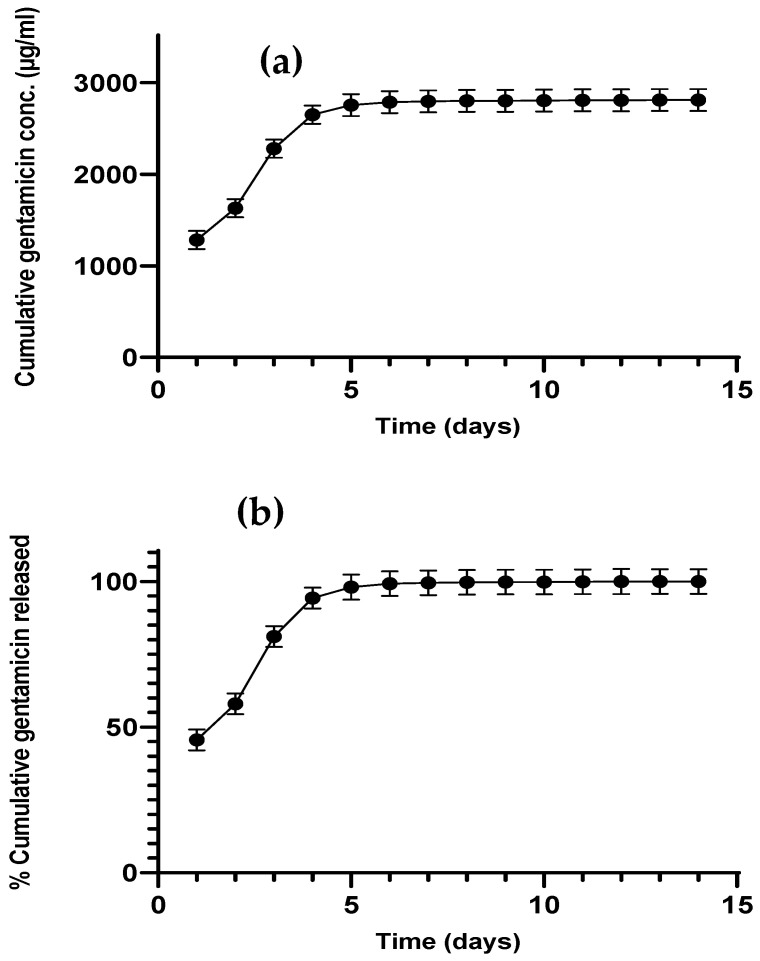
Gentamicin cumulative release in PBS media pH 7.4 from gentamicin-loaded CNT as concentration (**a**) or percentage release (**b**) (*n* = 3 ± SD).

More than 80 percent of the antibiotic was released in the first 5 days from gentamicin-loaded CNT, as shown in Figure 4a,b. After day 5, gentamicin release reached a plateau, where no significant increase in gentamicin release was observed over time. Gentamicin release from gentamicin-loaded CNT (before incorporation into the cement matrix) was 100%, because the release was not hindered by any surrounding matrix, and the hydrophilic drug escaped the hydrophobic CNT surface.

### 3.3. Gentamicin Release from Bone Cement

**Figure 5:**
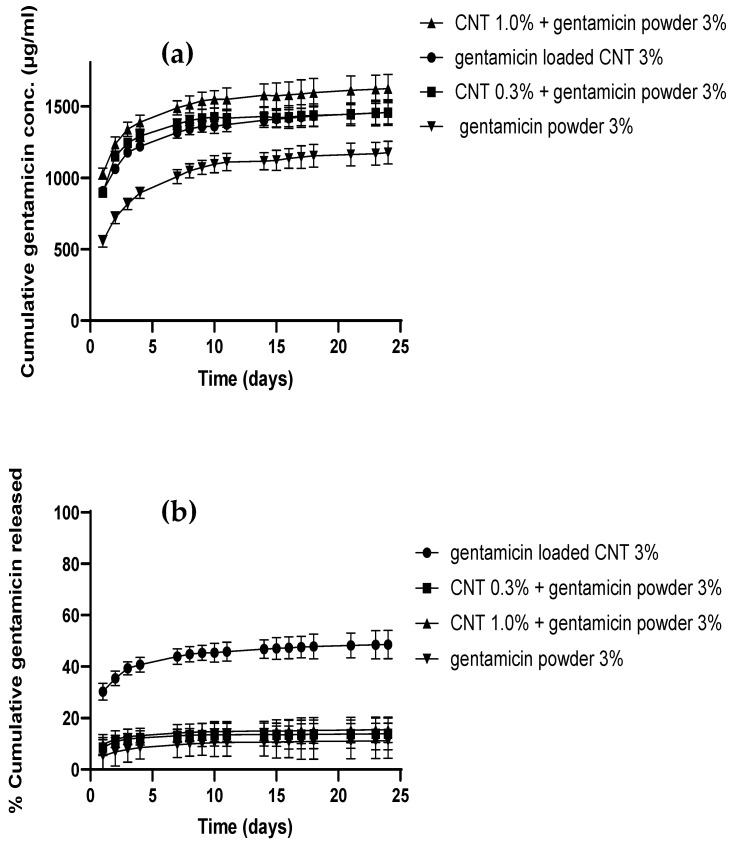
Gentamicin cumulative release profiles from different types of cement as concentration (**a**) and percentage (**b**) (*n* = 3 ± SD).

Gentamicin release from different cement types was studied at pH 7.4 in PBS media, which simulates the pH in healthy joints [36]. Figure 5 shows the cumulative gentamicin release from different bone cements as concentration or percentage for 25 days. All the CNT-containing bone cements released a significantly higher amount of gentamicin than the gentamicin powder containing cement (*p* < 0.05) (Figure 5a), while the CNT containing bone cements had a similar release profile above 1000 µg/mL (*p* > 0.05). In terms of percentage release, not all of the gentamicin added to the bone cement dough was released from the cement release samples (Figure 5b). The gentamicin-loaded CNT cement showed significantly higher percentage release (45%) than other types of bone cement (*p* < 0.05), which had nearly the same percentage of drug release (15%). However, the release continued for 25 days from all bone cement types tested at different concentrations and percentage levels according to the cement type. The presence of CNT in the cement enhanced gentamicin release in all cement types. Moreover, increasing CNT levels to 3% increased concentration and percentage released as observed in gentamicin-loaded CNT cement.

### 3.4. Mechanical Properties

**Figure 6:**
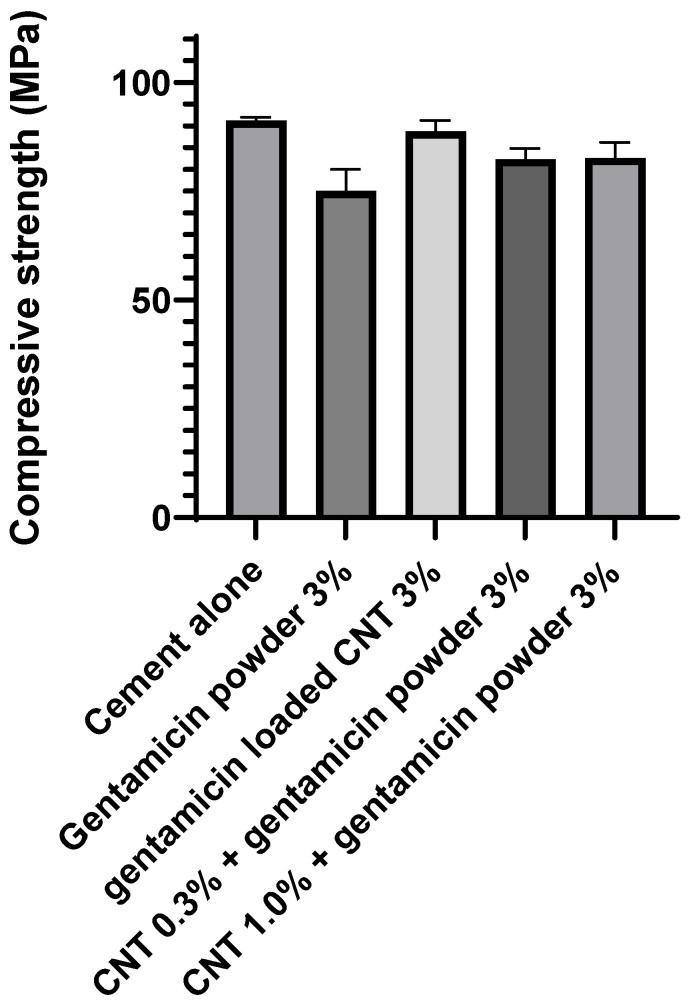
Compressive strength for different types of bone cement after aging at 37 °C for 3 months (*n* = 6 ± SD).

The CNT-containing cements had similar compressive strength compared to the gentamicin powder-containing bone cement (*p* > 0.05) (Figure 6).

### 3.5. Water Uptake Study

**Figure 7:**
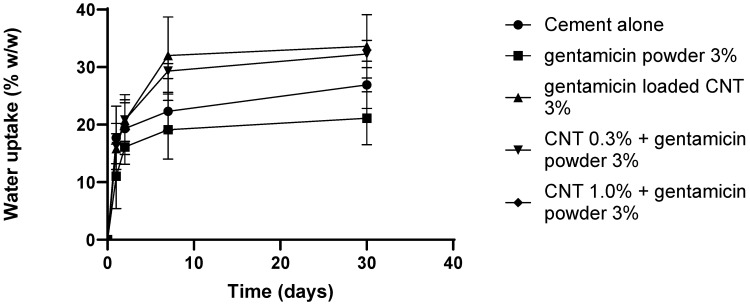
Water uptake for different bone cements after incubation in pH 7.4 PBS buffer (*n* = 3 ± SD).

The water uptake was comparable between different types of bone cement after 30 days of incubation (*p* > 0.05) (Figure 7). The cement samples gained weight in the first 7 days. After that, the weight of the samples and water content remained stable, where gentamicin powder cement had lower uptake than CNT-containing cements.

### 3.6. Antimicrobial Activity

**Figure 8:**
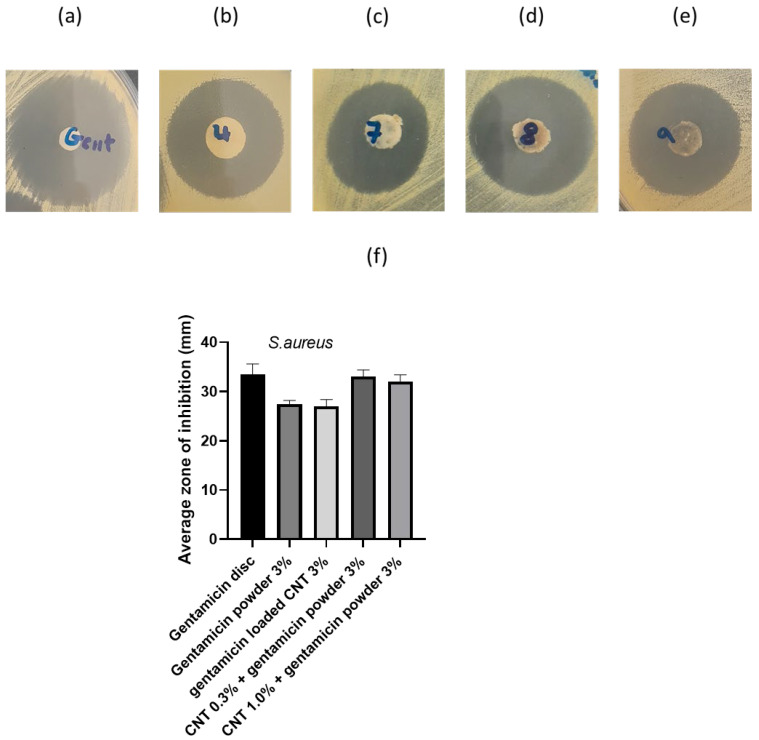
Zones of inhibition for *S. aureus*: (**a**) 10 μg gentamicin disc, (**b**) gentamicin powder 3%, (**c**) gentamicin-loaded CNT 3%, (**d**) CNT 0.3% + gentamicin powder 3%, (**e**) CNT 1% + gentamicin powder 3%, (**f**) average zone of inhibition (*n* = 3 ± SD).

Figure 8 displays the zones of inhibition and average radii attained from the agar diffusion assay. The gentamicin powder 3% and gentamicin-loaded CNT 3% cement demonstrated a lower zone of inhibition than the gentamicin disc (*p* < 0.05), while CNT 0.3% + gentamicin powder 3% and CNT 1% + gentamicin powder 3% had similar zones of inhibition to the gentamicin disc (*p* > 0.05). These findings highlight the availability of the incorporated gentamicin sulfate to inhibit *S. aureus* growth.

### 3.7. Cytotoxicity Analysis

MTT assay

**Figure 9:**
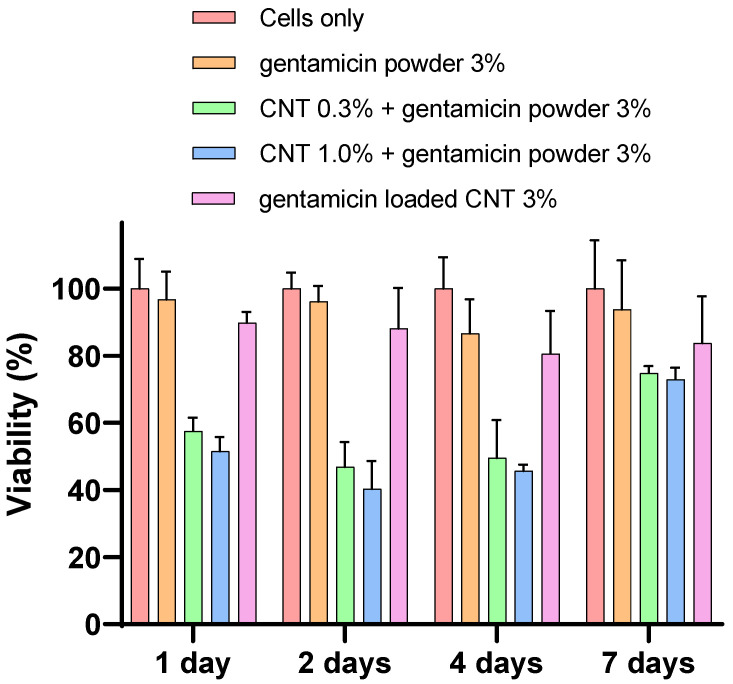
Mitochondrial activity (MTT assay) for osteoblasts with different types of cement (*n* = 6 ± SD).

The mitochondrial activity showed no significant difference between 0.3 and 1% CNT-containing bone cement in each time point (days 1, 2, 4, and 7) nor with 3% CNT bone cement at days 4 and 7 (*p* > 0.05) (Figure 9). However, the gentamicin powder-containing cement without CNT had higher mitochondrial activity than 0.3 and 1% CNT-containing bone cement in day 1, 2, and 4 (*p* < 0.05). All cements tested had similar mitochondrial activity at day 7.

LDH assay

**Figure 10:**
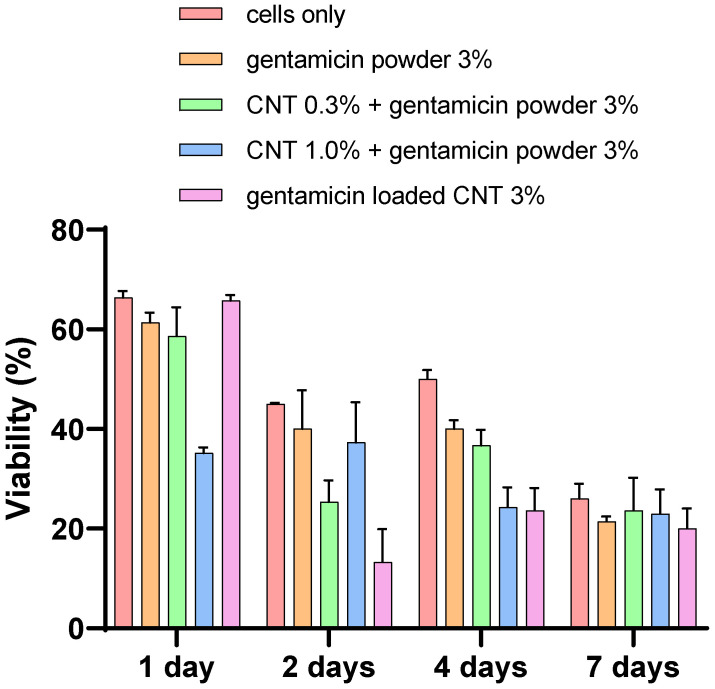
Cytocompatibility test (LDH assay) for osteoblasts with different cements types (*n* = 6 ± SD).

The viability of the cement gentamicin powder-containing bone cement was higher than other gentamicin-loaded CNT 3% bone cement at days 2 and 4 (*p* < 0.05) (Figure 10). However, the viability was the same for all different types of cement at day 7 (*p* > 0.05). The CNT-containing bone cements showed variable viability in days 1, 2, and 4, reaching the lowest viability level of less than 30% on day 7.

Alizarin red

**Figure 11:**
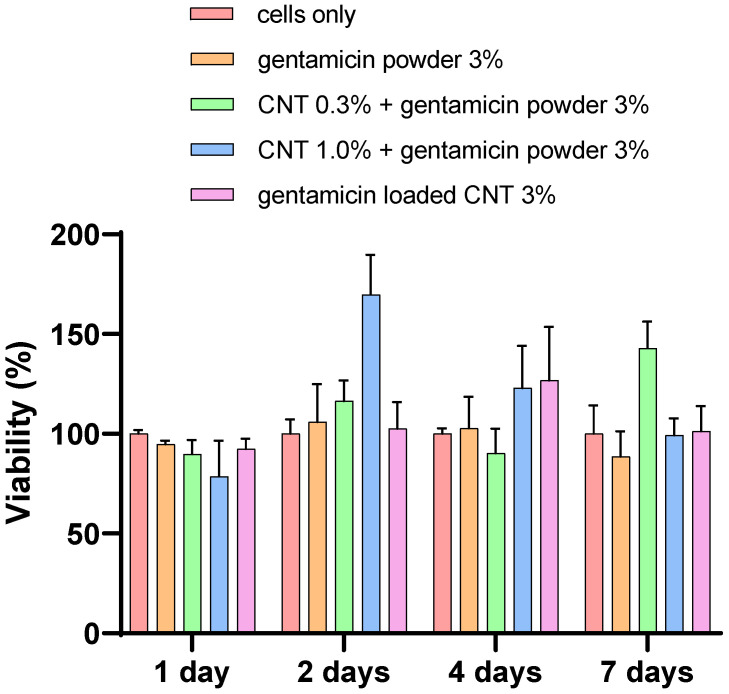
Cytocompatibility test (alizarin red assay) for different osteoblasts with different types of cement (*n* = 6 ± SD).

**Figure 12:**
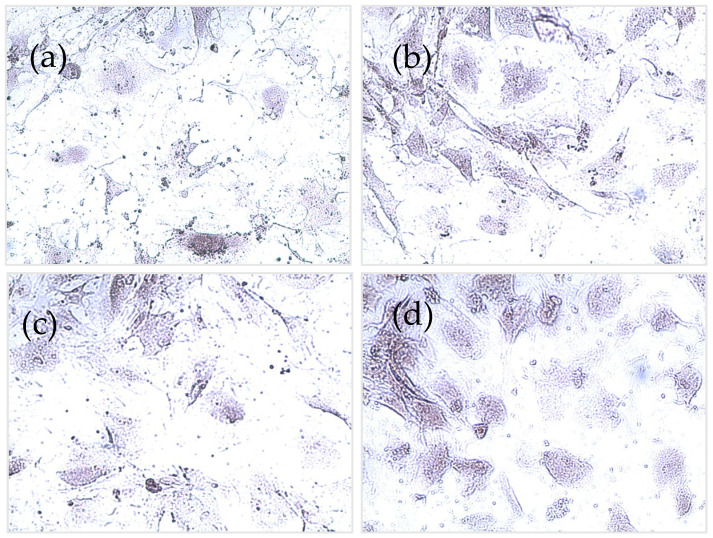
Alizarin red images for (**a**) cement only, (**b**) 0.3% CNT, (**c**) 1% CNT, and (**d**) 3% gentamicin-loaded CNT.

Production of calcium from osteoblasts was not significantly different from all types of bone cement (*p* > 0.05), with the presence of CNT or not (Figure 11). However, calcium production was increased at each time point, reaching the highest levels at day 7 in all types of cement. Figure 12 shows stained images with alizarin red for different types of bone cement at day 7.

## 4. Discussion

TJR is the last choice for the treatment of end-stage osteoarthritis patients to retrieve their mobility. This procedure is performed increasingly in patients suffering from osteoarthritis with obesity and age as risk factors [37]. ALBCs are typically used to fix the implant in place and release antibiotics, preventing PJIs. Novel approaches for prolonging antibiotic release from the bone cement after the surgery are urgently needed for fighting the life-threatening implications of PJIs [38]. This study aimed to explore the incorporation of CNT into the bone cement for sustaining antibiotic release without negatively affecting the cement properties. Therefore, different types of bone cement (different compositions of CNT/gentamicin) were prepared and characterized with different concentrations of CNT and gentamicin.

In vitro release studies were conducted to study gentamicin release from commercial gentamicin powder and CNT-containing bone cements at pH 7.4, which represents healthy joints before infection-induced acidosis occurs [39,40]. Sink conditions were attained by maintaining the highest concentration in all release samples below the gentamicin solubility concentration, which is more than 10 mg/mL in PBS. The CNT-containing bone cements exhibited prolonged release at a higher concentration than the commercial (gentamicin powder cement, Figure 5), despite having an equivalent amount of gentamicin or less (as in the case of gentamicin-loaded CNT). The reason for this behavior is that gentamicin needs to be released first from the CNT before the migration from the cement matrix. Gentamicin is a small molecule with higher diffusivity through PMMA, giving burst release in the first few days, which gradually slows down over a longer time. The presence of CNT at 1% concentration gave the highest concentration profile, while gentamicin-loaded cement had a higher percentage of drug release. These results could be attributed to the homogenous distribution of CNT, which could facilitate the diffusion of gentamicin by forming a nano-channel network [17,21]. Many studies have shown that less than 10% of the added antibiotic is released from the PMMA cement [15,16], which is caused by the antibiotic being deeply entrapped in the cement matrix. Moreover, a low percentage of drug released could be attributed to the inactivation of the drug during the bone cement setting free radical polymerization reaction [10,17]. Therefore, the encapsulation of the drug in nanocarrier systems could protect the antimicrobial drug, resulting in greater release yield and reduced drug loss, such as liposomes or silica nanocarriers [17,19], explaining the release observed in Figure 5. The release profiles from encapsulated systems incorporated into PMMA bone cement usually follow first release kinetics [41]. The release of gentamicin from CNT nanoparticles (Figure 4) alone was faster than PMMA bone cement, as the cement matrix further slowed down drug release. Thus, drug release from the cement matrix is the rate-limiting step because complete release from CNT needs 7 days, compared to 25 days from bone cement. The presence of CNT in the bone cement increased the percentage of drug released as observed in gentamicin-loaded CNT 3%, where 45% of the drug was released compared to 15% drug release from commercial gentamicin powder cement (Figure 5). This observation agrees with previous studies on antibiotic elution from PMMA bone cement [15,16], which can be explained by drug entrapment inside the PMMA composite stopping the migration of the drug from bulk to the cement surface.

In the literature, different plating protocols are employed to assess the antimicrobial activity of bone cement, because there is no standardized method for assessing the antimicrobial properties of bone cement. For example, Berchert et al., 2000 studied the duration of the lag phase between different treatments [42], while Ayer et al. (2016) measured the zones of inhibition in liposomal formulations using the agar diffusion test [19]. In this study, the agar diffusion test confirmed the bioavailability of the encapsulated gentamicin in the CNT containing bone cement, as obtained from the reproducible zones of inhibition (Figure 8). Consistent shape and size between different CNT and powder-containing cement suggest good gentamicin dispersion on the cement surface, resulting in even diffusion through agar. The inhibition zones were higher in some CNT-containing bone cements because of higher gentamicin concentrations. In this study, the antimicrobial efficacy was only tested against one type of bacterial strain (*Staphylococcus aureus*) as an indicator of antimicrobial activity of the developed CNT-containing nanocomposites compared to the gentamicin powder-containing cement. However, other studies tested the antimicrobial activity for different microbial strains in PJIs, including: coagulase-negative Staphylococci, Streptococci, Gram-negative bacilli (i.e., *Escherichia coli* and *Pseudomonas aeruginosa*), anaerobes, and Enterococci [43].

The compressive strength of bone cement was not influenced by the presence of the CNT (Figure 6). Many studies in the literature reported that the addition of CNT to bone cement improved the mechanical properties [44,45,46]. Carbon nanotubes are known for a more significant aspect ratio and higher modulus [47]. A study reported that the tensile strength of polypropylene fibers reinforced with CNT was increased by 40% [48]. CNT/PMMA composites prepared by melting blending were well dispersed in the nanocomposite, and the storage modulus of the cement was significantly increased [44]. In another study, a polymer extrusion technique was used to prepare CNT mixed in a PMMA matrix and concluded that the cement strength was significantly enhanced by even small amounts of CNTs [46]. This could be explained by strong combining interface formation between PMMA and CNT caused by CNT participation in the PMMA free radical polymerization reaction [45].

Bone cement fluid absorption inside the body affects the mechanical and surface properties, resulting in a molecular weight decrease over time [49]. Therefore, determining the initial amount of water uptake is crucial for studying the physicochemical characteristics of bone cement. Furthermore, the increased water uptake at physiological conditions negatively affects the mechanical strength, because of the water plasticizing effect that minimizes the attraction and increases flexibility between polymeric chains [50]. In this study, bone cement samples had comparable water uptake in the first 7 days (Figure 7), when in contact with PBS, after which gentamicin powder containing cement had lower uptake than the others. The higher uptake for the CNT-containing cements can be attributed to the possible formation of microchannels during the cement setting reaction, allowing more PBS absorption.

Device osseointegration and osteoblast growth on the PMMA cement are needed for having a successful TJR. However, there are always concerns about PMMA osseointegration and cytocompatibility, as it does not provide the optimal substrate characteristics that support cell growth [51]. However, bone cement is routinely used in clinical practice as it provides sufficient cytocompatibility. Nano-hydroxyapatite offers biocompatibility and has many dental applications because of its resemblance to the nonorganic bone structure [52]. However, nano-hydroxyapatite does not have enough mechanical properties for application in high load bearing joint such as in TJR [21]. The response of osteoblast cells when in contact with PMMA is important for the assessment of novel bone cements [52]. In this study, the viability and mineralization of cells were not affected by the presence of CNT in the cement after 7 days of incubation (Figure 9, Figure 10, Figure 11 and Figure 12). To validate and assess different variables, our experiments employed a range of cytotoxicity assays (MTT, LDH, alizarin red, and imaging). For example, MTT assesses mitochondrial activity, LDH assesses cell membrane integrity, alizarin red measures calcium production, and imaging evaluates cell morphology. Shen et al. (2016) reported that 5.3% CNT incorporation into bone cement resulted in 85% cell viability when tested on 3T3 mouse fibroblast cells [21]. Orsmby et al. (2012) studies viability, cell adhesion, and morphology for the effect of CNT PMMA nanocomposites in contact with MG-63 cells [53]. The study concluded that PMMA cement containing 1% CNT showed the best cell adhesion without significant differences in cell morphology and viability at day 7. Wang et al. (2019) reported a significant enhancement in the integration between cement and bone in an animal bone defect model (New Zealand rabbit) at a 1% CNT level, leading to a 42.2% bone ingrowth ratio after 12 weeks of implantation [54]. This finding suggests that the osteointegration and cytocompatibility of the cement can be improved by adjusting CNT loading. In summary, our in vitro results presented that the developed CNT PMMA cement is safe to use.

## 5. Conclusions

CNT nanoparticles were successfully incorporated in PMMA bone cement loaded with gentamicin sulphate antibiotic. The developed nanocomposite displayed prolonged-release that continued for 25 days, as observed in gentamicin-loaded CNT 3%, where 45% of the drug was released compared to 15% drug release from commercial gentamicin powder cement. Furthermore, the CNT containing nanocomposite preserved the antimicrobial activity as demonstrated by the agar diffusion test. Additionally, the mechanical properties of the nanocomposites were not adversely affected by the presence of CNTs in the nanocomposites, as displayed by compressive strength testing. Finally, CNT-containing nanocomposites showed cytocompatibility towards differentiated human osteoblast cells, as demonstrated by MTT, LDH, alizarin red, and microscopy. Therefore, the developed CNT nanocomposites may serve as a novel PMMA bone cement for the treatment and prophylaxis from PJIs.

## Figures and Tables

**Table 1:** Specific characterization of CNTs (Sigma-Aldrich code: 755125, Nanocyl Inc., Belgium) and methods of measurements.

Property	Unit	Value	Method of Measurement
Average diameter	10^−9^ m	9.5	TEM
Average length	μm	1.5	TEM
Carbon content	%	>87	TGA (thermal gravimetric analyzer)
Surface area	m^2^/g	500	BET (Brunauer, Emmett, and Teller)
–COOH groups (surface modification)	%	10	XPS (X-ray photoelectron spectroscopy)

## Data Availability

The data presented in this study are available upon request from the corresponding author.
